# Supplementary material for: Drivers and distribution of global ocean heat uptake over the last half century
Source: Nat Commun. 2022 Sep 7;13:4921. doi: 10.1038/s41467-022-32540-5 (PMC9452516; doi:10.1038/s41467-022-32540-5)
Supplement: Supplementary file 1 — Supplementary Information [file 41467_2022_32540_MOESM1_ESM.pdf]

Supplementary Information for

Drivers and distribution of global ocean heat uptake over the last half century

Maurice F. Huguenin[1,2,3], Ryan M. Holmes[1,2,4,5] and Matthew H. England[1,3]

[1]Climate Change Research Centre, University of New South Wales, Sydney, New South Wales, Australia

[2]ARC Centre of Excellence in Climate Extremes, University of New South Wales, Sydney, New South Wales, Australia

[3]ARC Australian Centre for Excellence in Antarctic Science, University of New South Wales, Sydney, New South Wales, Australia

[4]School of Mathematics and Statistics, University of New South Wales, Sydney, New South Wales, Australia

[5]School of Geosciences, University of Sydney, Sydney, New South Wales, Australia

Corresponding author: Maurice F. Huguenin (m.huguenin-virchaux@unsw.edu.au)

Contents:

- Supplementary Figures 1 - 9
- Supplementary Tables 1 - 2
- Supplementary References

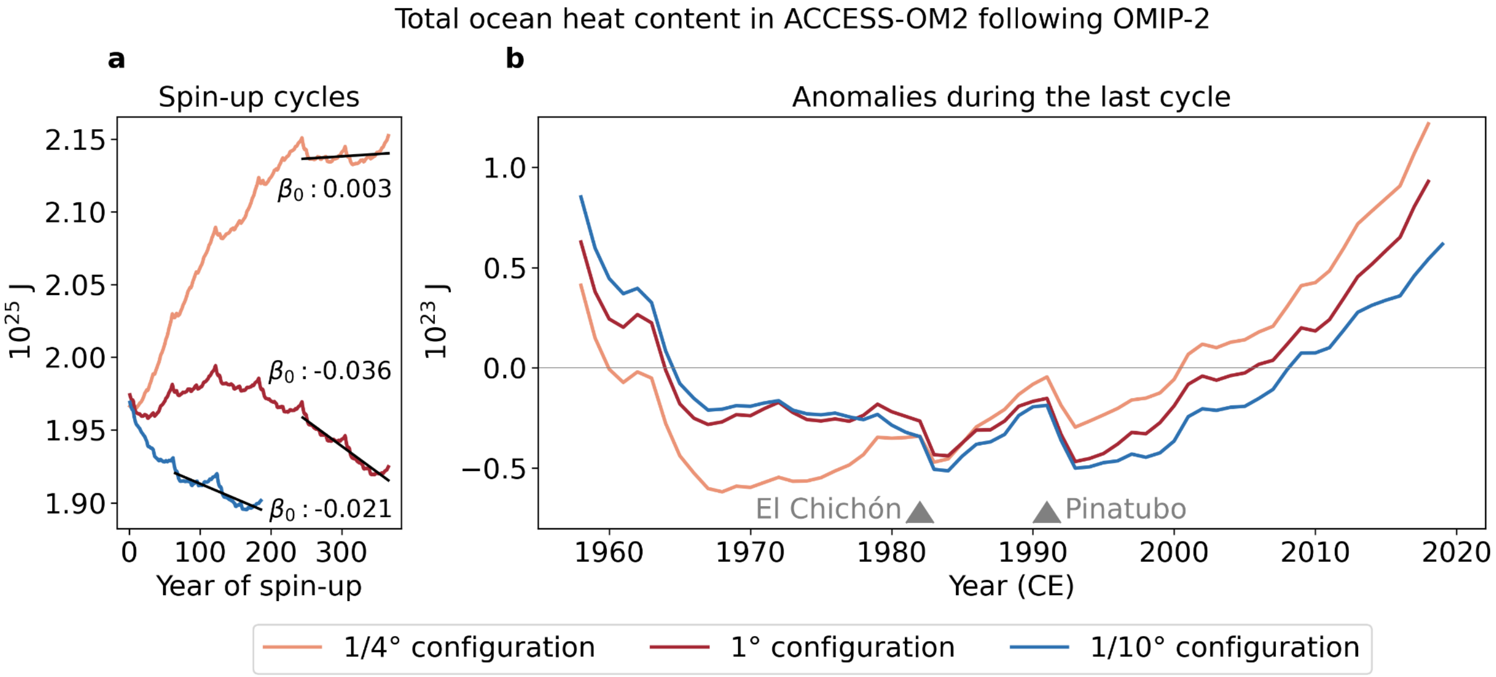

Supplementary Fig. 1. Total ocean heat content anomalies in ACCESS-OM2. a, Total ocean heat content (10^{25} J) in the 1°, 1/4° and 1/10° model configuration following the OMIP-2 protocol. Only three spin-up cycles are available in the 1/10° model due to the cost involved in running this configuration. In black the linear trends over the last two cycles with the trend values β_0 in units of 10^{25} J century^{-1}. **b**, Anomalies for the three configurations of the model during the last cycle, calculated by removing the respective linear trend over the last two cycles in **a**.

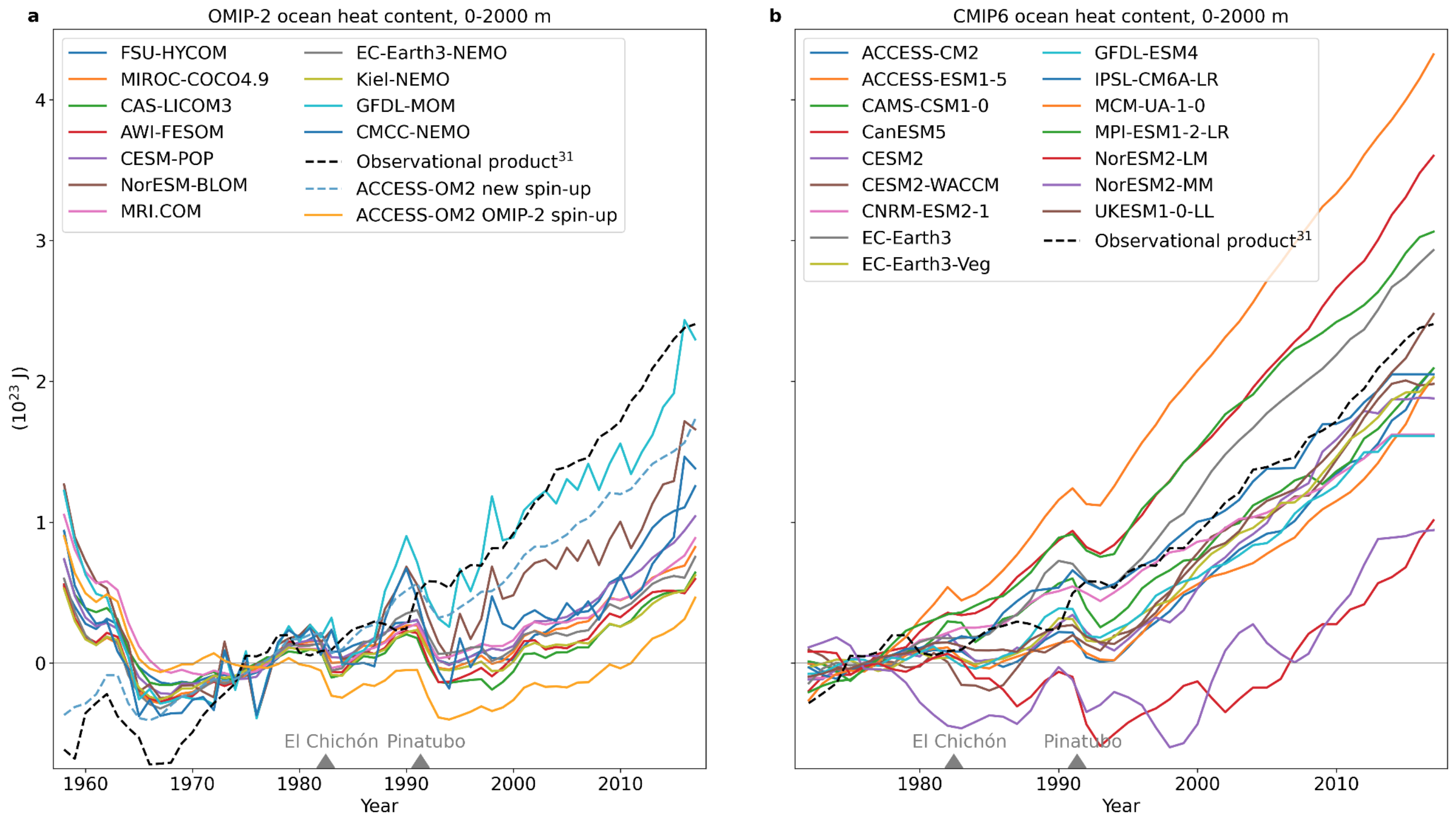

Supplementary Fig. 2. Simulated global ocean heat content in OMIP-2 and CMIP6. a, Time series of 1958-2017 OHC anomalies in the eleven OMIP-2 models presented in Tsujino et al. (2020)[49] and in ACCESS-OM2. The observations from Levitus et al. (2012)[31] are shown as the dashed black line. The new simulation with full interannual forcing is shown as the dashed blue line. The anomalies are calculated by first removing the linear trend over the last two spin-up cycles and then removing the mean of the 1972-1981 baseline period. **b**, As in **a** but for 25 CMIP6 models[25] over 1972-2017.

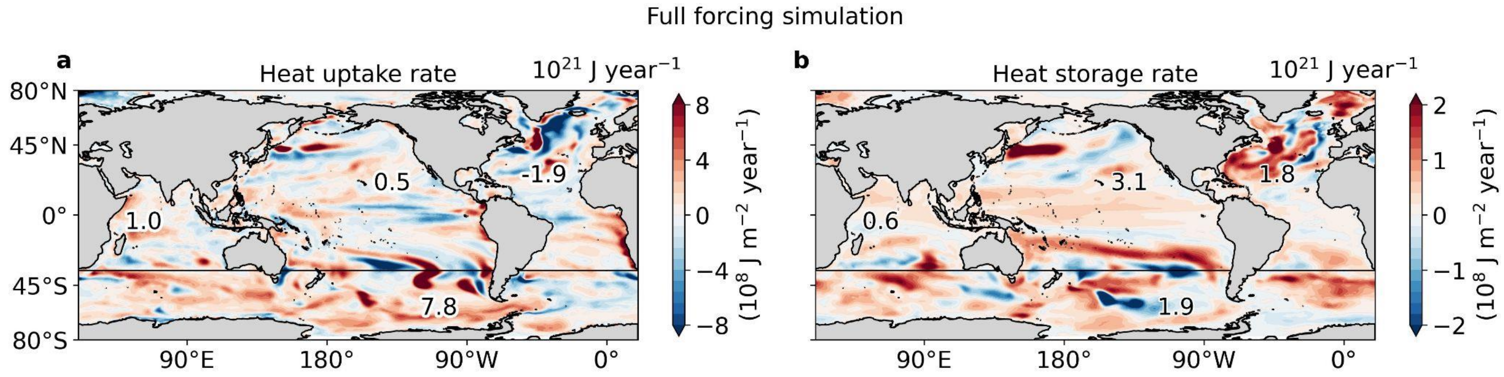

Supplementary Fig. 3. Spatial distribution of ocean heat uptake and storage trends over 1997-2017 in the simulation with full interannual forcing. **a**, Time-integrated net surface heat flux anomalies (10^8 J m^{-2} year^{-1}) with positive heat uptake defined as into the ocean. The basin-wide values (10^{21} J year^{-1}) show the total area-integrated trends over a particular ocean basin with the boundaries set by the blue lines. The Southern Ocean ends at 36°S, the Bering Strait is at 65°N and the Indonesian Throughflow is defined between Java, New Guinea (105°W to 134°W) at 3°S and the Australian continent (20°S to 6°S) at 137°W. The Atlantic Ocean contributions include the Arctic Ocean north of 65°N and the marginal Hudson Bay, Baltic and Mediterranean basins. The Indian Ocean component also includes the Red Sea. The basin-wide values are rounded to one-decimal point accuracy. **b**, As in **a** but for the heat storage trends.

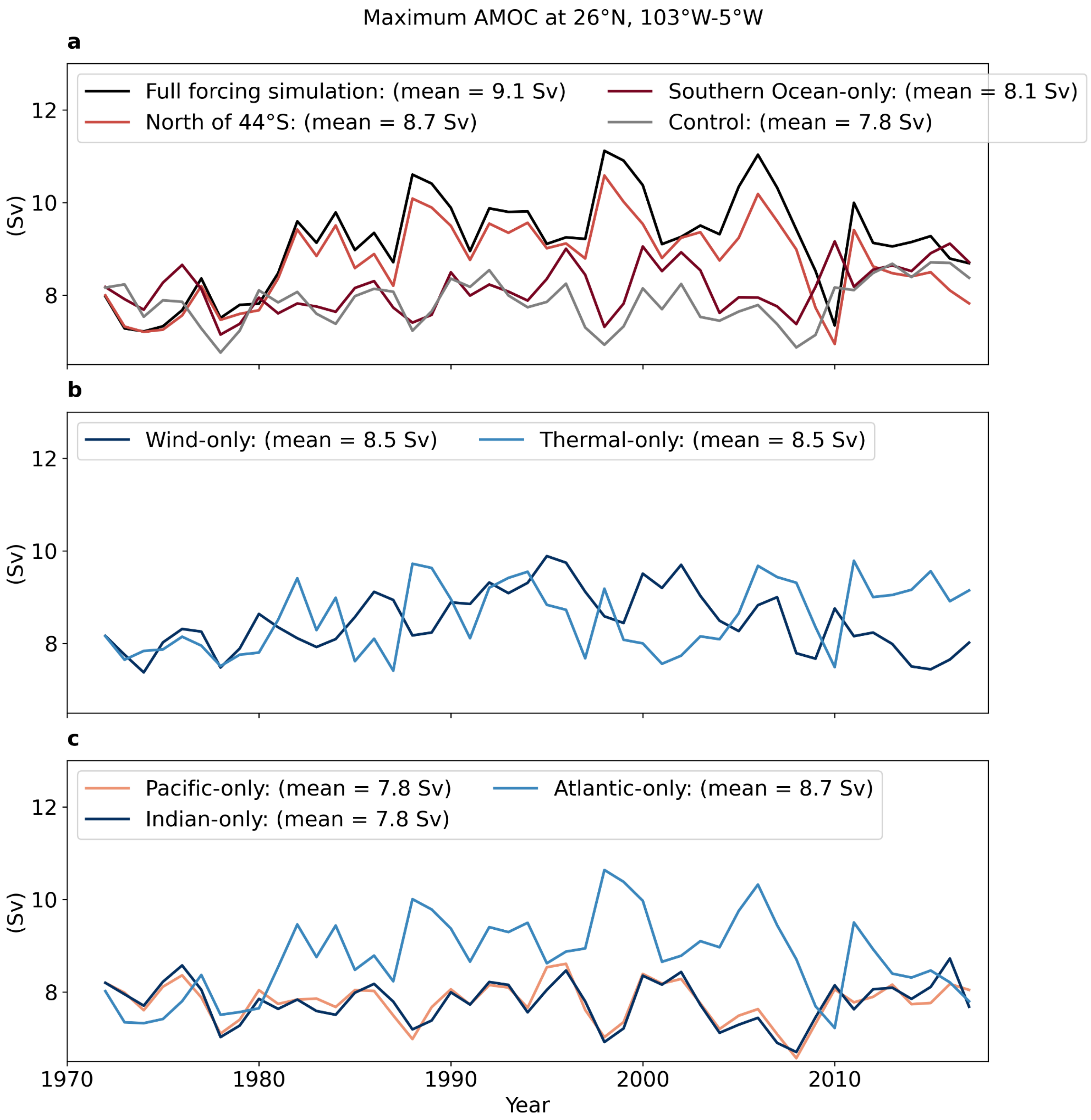

Supplementary Fig. 4. Maximum strength of the Atlantic Meridional Overturning (AMOC) Streamfunction at 26°N between 103°W and 5°W. a, Annual mean upper overturning cell magnitude as a function of time (Sv, 1 Sv = 10^6 m^3 s^{-1}), defined as the maximum value of the global overturning streamfunction computed on density surfaces,

measured at 26° N, integrated between 103° and 5°W and for potential density classes that exceed 1035.5 kg m^{-3}. Time series are shown for the full forcing simulation as well as the control simulation, the Southern Ocean-only and the North of 44°S experiments. The mean AMOC strength in these experiments over the full time period is given as the mean for each time series rounded to one decimal point accuracy. **b, c** As in **a** but for the wind- and thermal-only experiments as well as the basin-wide Pacific-, Indian- and Atlantic-only experiments.

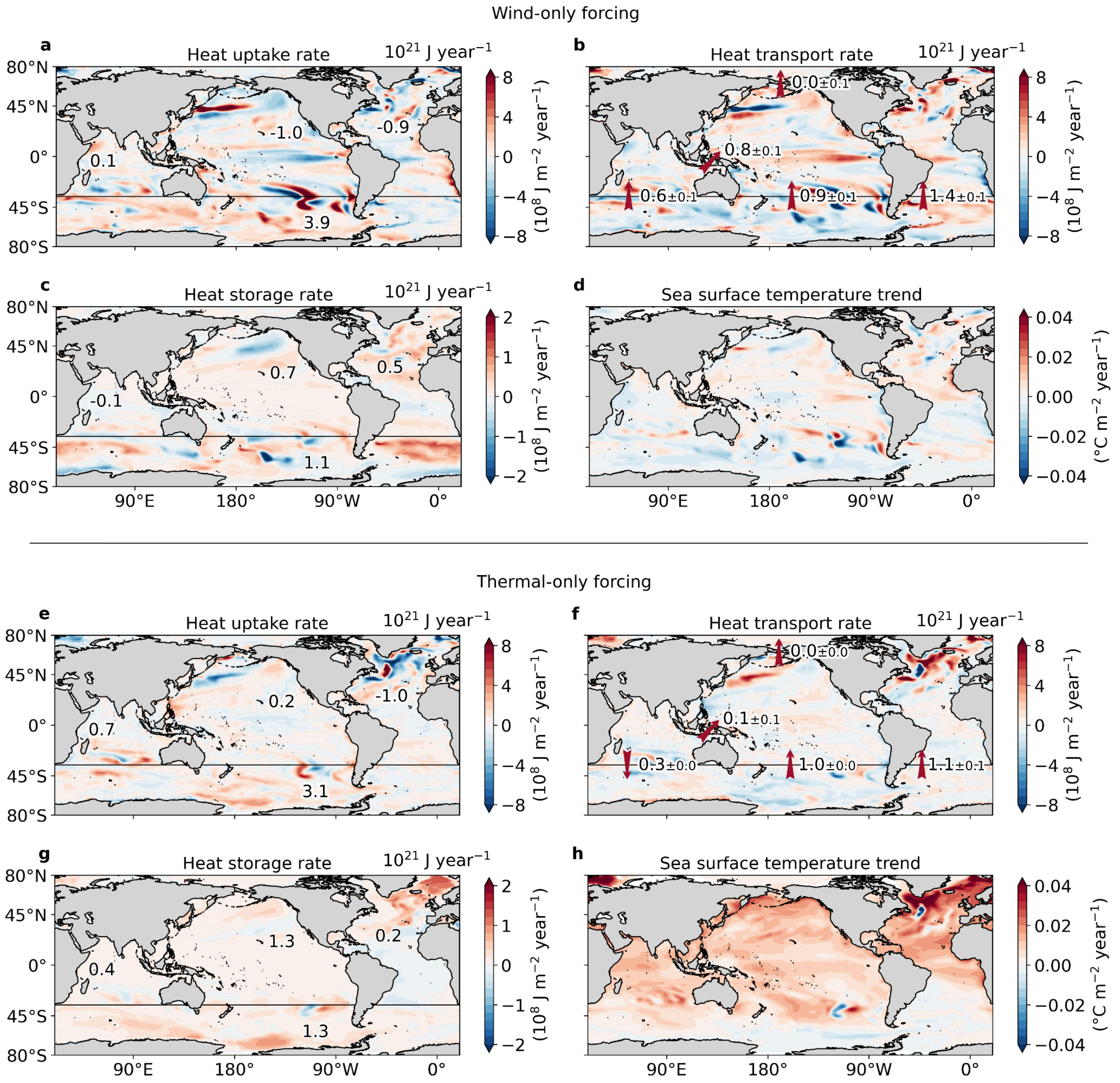

Supplementary Fig. 5. Spatial distribution of ocean heat uptake, transport, storage and sea surface temperature trends over 1972-2017 in the wind-only and thermal-only forcing experiment. a, Time-integrated net surface heat flux anomalies (10^8 J m^{-2} year^{-1}) with positive heat uptake defined as into the ocean. The basin-wide values (10^{21} J year^{-1}) show the total area-integrated trends over a particular ocean basin with the boundaries set by the

black lines across the Southern Ocean, the Indonesian Throughflow, the Bering Strait and the continental land masses. The Southern Ocean ends at 36°S, the Bering Strait is at 65°N and the Indonesian Throughflow (ITF) is defined between Java, New Guinea (105°W to 134°W) at 3°S and the Australian continent (20°S to 6°S) at 137°W. The Atlantic Ocean contributions include the Arctic Ocean north of 65°N and the marginal Hudson Bay, Baltic and Mediterranean basins. The Indian Ocean component also includes the Red Sea. The basin-wide values are rounded to one-decimal point accuracy. **b**, Anomalous heat transport convergence calculated as a residual from the **a** heat uptake and **c** heat storage (10^8 J m^{-2} $year^{-1}$). The anomalous heat transport rates and their uncertainties across transects (10^{21} J m^{-2} $year^{-1}$) are calculated from anomalous heat and volume transports (see Methods). **d**, Simulated SST trends (°C m^{-2} $year^{-1}$). Grid cells in **d** that have a climatological sea ice coverage above 85% have been removed and are shaded white. **e-f**, As in **a-d** but for the perturbation experiment where only atmospheric trends in thermal properties are applied (surface air temperature, humidity, radiation, precipitation and sea level pressure trends, while repeat decade forcing is used for the other forcing fields.

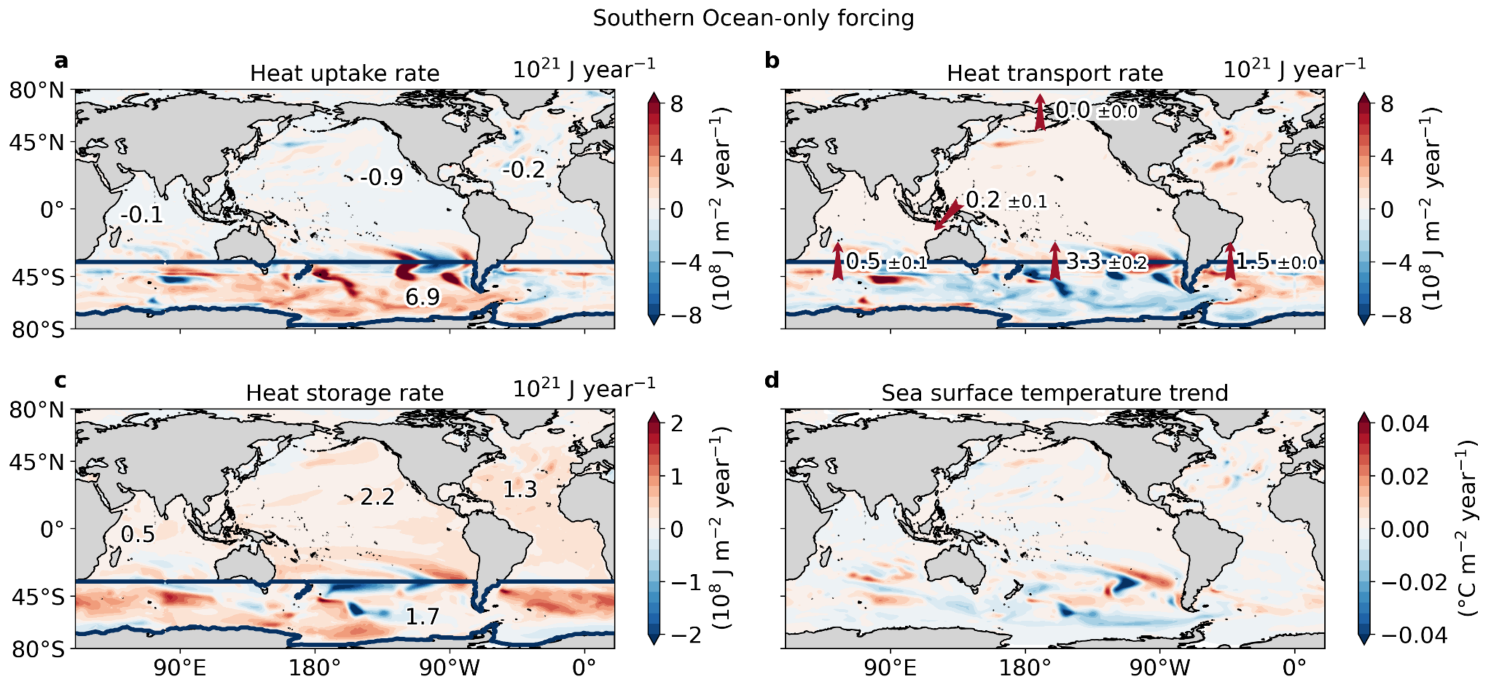

Supplementary Fig. 6. As in Supplementary Fig. 5 but for the Southern Ocean experiment where interannual forcing is applied south of 44°S over the region with blue outlines. Repeat decade forcing is applied north of 40°S and the tapering zone between the two forcing fields is from 44°S to 40°S.

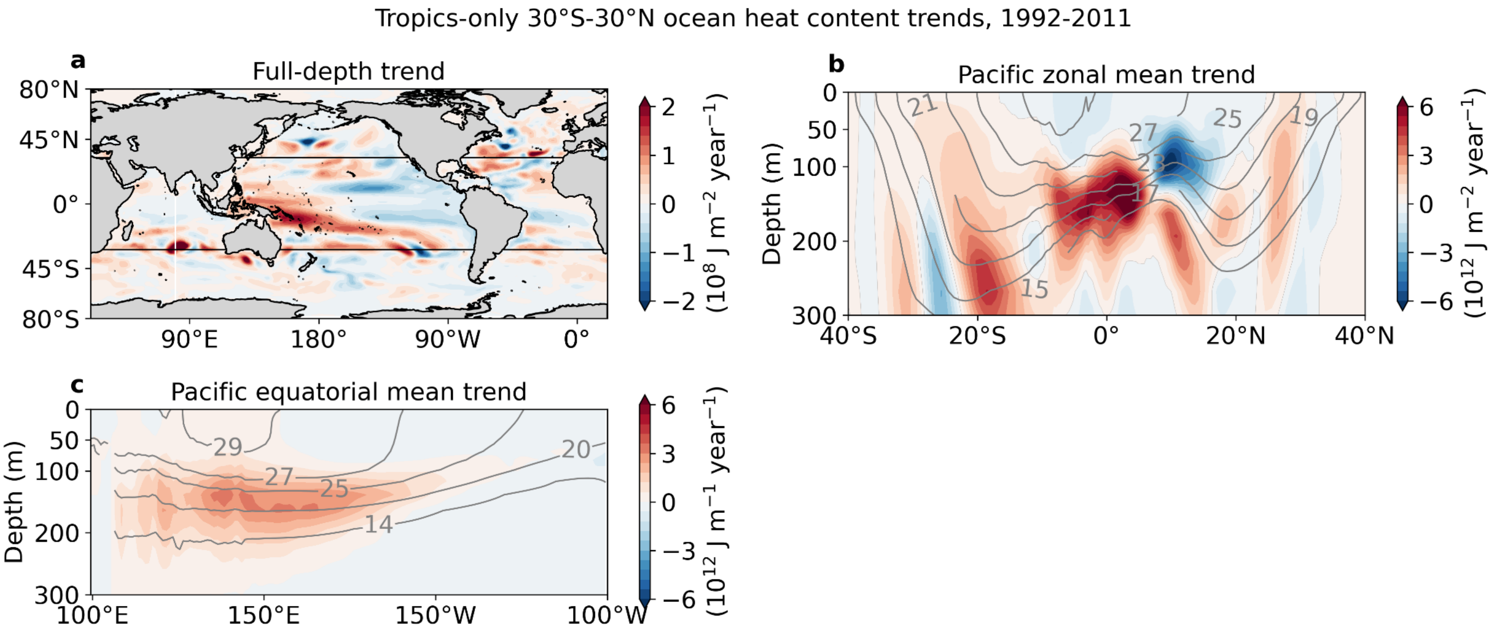

Supplementary Fig. 7. Ocean heat content trends over 1992-2011 in the Tropics-only 30°S-30°N experiment. a, Spatial pattern of anomalous ocean heat content trend over 1992-2011 (10^8 J m^{-2} $year^{-1}$). The horizontal blue lines indicate the region over which interannual forcing is applied (30°S-30°N). **b**, Zonal mean ocean heat content trends in the Pacific Ocean 100°E-100°W (10^{12} J m^{-2} $year^{-1}$). The contours show the climatological isotherms across the Pacific 40°S-40°N (°C). **c**, Equatorial mean 5°S-5°N ocean heat content trends in the Pacific Ocean (10^{12} J m^{-2} $year^{-1}$). The contours show the climatological isotherms across the Pacific 100°E-100°W (°C).

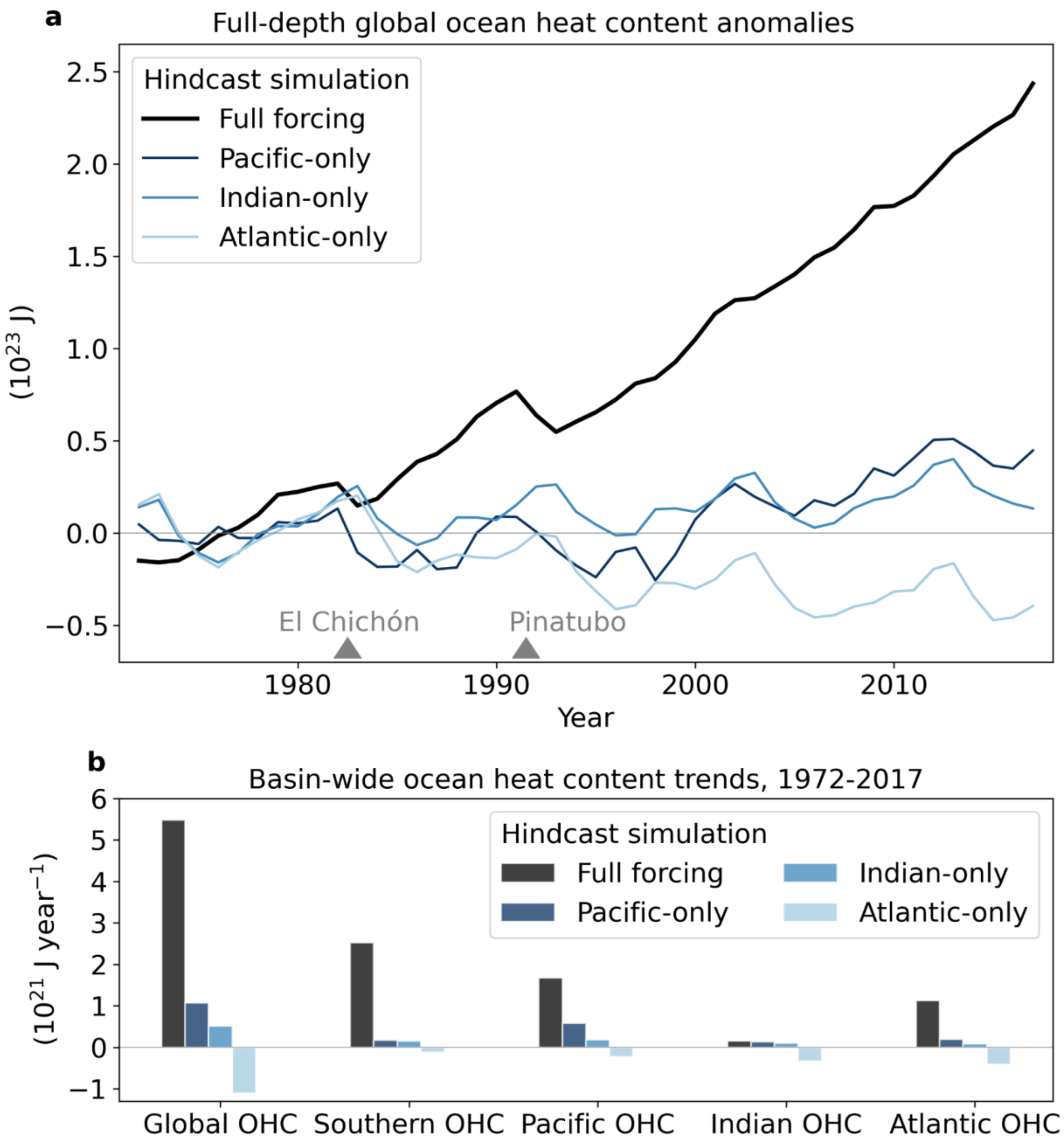

Supplementary Fig. 8. Ocean heat content changes since 1972 in the basin-wide simulations. a, Time series of the full-depth ocean heat content anomalies where interannual forcing is applied only over the Pacific, Indian and Atlantic basins compared with the simulation where interannual forcing is applied over the full ocean area (10^{21} J year^{-1}). For the Pacific Ocean simulation, we choose the southern interannual forcing/repeat decade forcing boundary at 44°S as this latitude marks the poleward extent of the shallow subtropical cells. For the Indian and Atlantic Ocean simulations, we set the boundary to 35°S at the southern tip of Africa. **b**, The basin-integrated ocean heat content trends for the perturbation experiments in **a** with the boundaries set by the dark blue lines in Fig. 2 in the main manuscript.

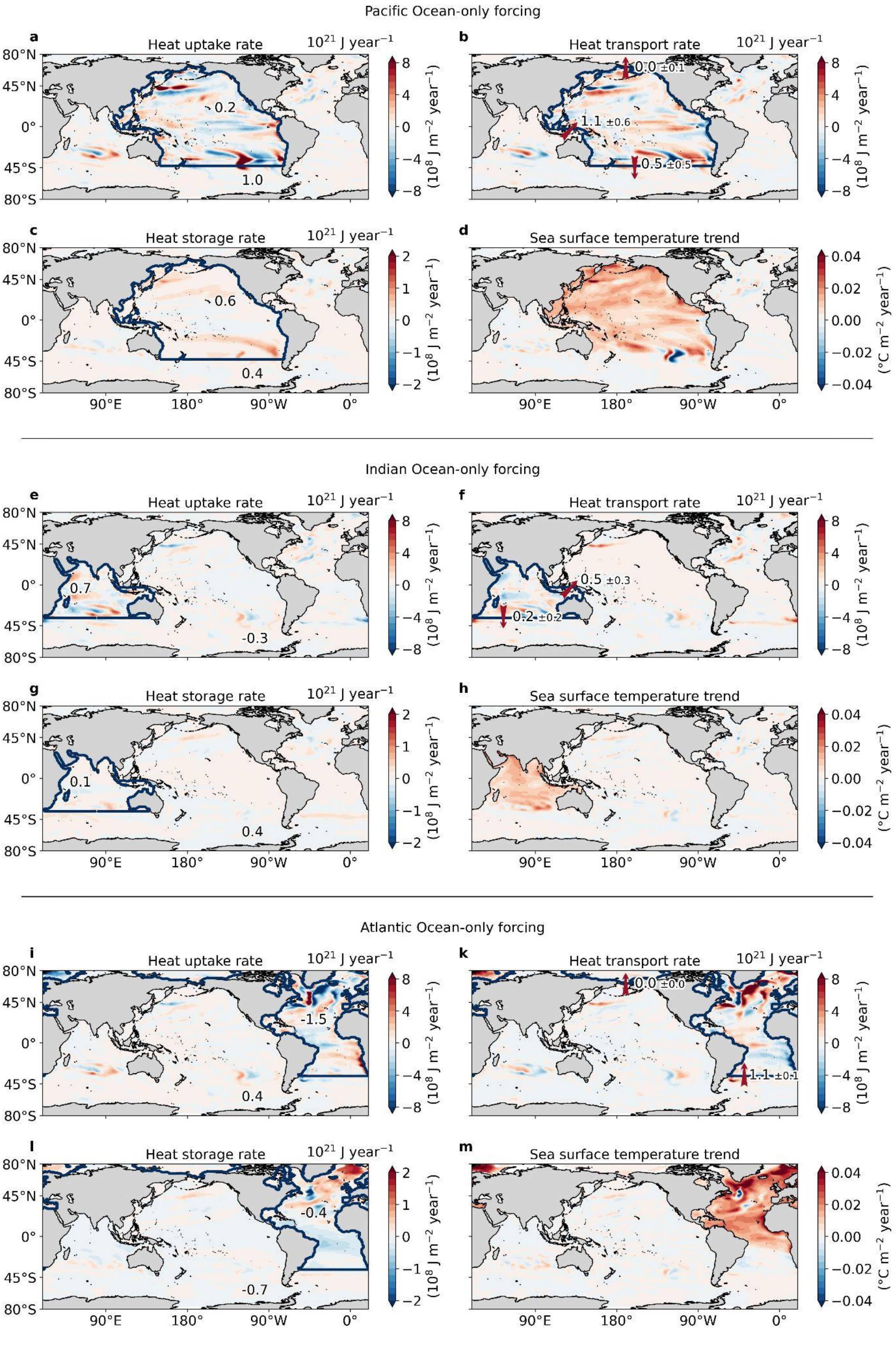

Pacific Ocean-only forcing
a
Heat uptake rate
10^21 J year^-1
0.2
1.0
b
Heat transport rate
10^21 J year^-1
0.0 ±0.1
1.1 ±0.6
0.5 ±0.5
c
Heat storage rate
10^21 J year^-1
0.6
0.4
d
Sea surface temperature trend
Indian Ocean-only forcing
e
Heat uptake rate
10^21 J year^-1
0.7
-0.3
f
Heat transport rate
10^21 J year^-1
0.5 ±0.3
0.2 ±0.2
g
Heat storage rate
10^21 J year^-1
0.1
0.4
h
Sea surface temperature trend
Atlantic Ocean-only forcing
i
Heat uptake rate
10^21 J year^-1
-1.5
0.4
k
Heat transport rate
10^21 J year^-1
0.0 ±0.0
1.1 ±0.1
l
Heat storage rate
10^21 J year^-1
-0.4
-0.7
m
Sea surface temperature trend
(10^8 J m^-2 year^-1)
(°C m^-2 year^-1)
80°N
45°N
0°
45°S
80°S
90°E
180°
90°W
0°

Supplementary Fig. 9. As in Supplementary Fig. 5 but for the **a-d** Pacific Ocean-only, **e-h** Indian Ocean-only and **i-m** Atlantic Ocean-only forcing experiment. The blue outlines indicate the region over which interannual forcing is applied while repeat decade forcing is used over the remaining ocean area.

	Net shortwave heat flux	Net longwave heat flux	Sensible heat flux	Latent heat flux
Full forcing simulation	0.1	3.1	3.3	-0.3
Wind-only forcing	0.6	1.9	3.7	-2.7
Thermal-only forcing	0.1	3.0	2.4	-3.1

Supplementary Table. 1. Heat flux contributions to anomalous Southern Ocean heat uptake. Contributions of the basin-wide integrated heat flux trends over the Southern Ocean south of 36°S in the simulation with full interannual forcing, with wind-only forcing and thermal property-only forcing (10^{21} J $year^{-1}$). The values are rounded to one-decimal point accuracy. Only the four major heat flux components are shown here without the surface flux contributions from surface volume exchanges and heat exchanges with sea ice.

Model acronym	Ensemble member	Reference
ACCESS-CM2	r1i1p1f1	Dix et al. (2019a, 2019b, 2019c)
ACCESS-ESM1-5	r1i1p1f1	Ziehn et al. (2019a, 2019b, 2019c)
CAMS-CSM1-0	r1i1p1f1	Rong (2019a, 2019b, 2019c)
CanESM5	r1i1p1f1	Swart et al. (2019a, 2019b, 2019c)
CESM2	r1i1p1f1	Danabasoglu (2019, 2019a, 2019b)
CESM2-WACCM-FV2	r1i1p1f1	Danabasoglu (2019c, 2019d, 2019e)
CESM2-WACCM	r1i1p1f1	Danabasoglu (2019f, 2019g, 2019h)
CNRM-ESM2-1	r1i1p1f2	Seferian (2018a, 2018b), Voldoire (2019)
EC-Earth3	r1i1p1f1	EC-Earth (2019a, 2019b, 2019c)
EC-Earth3-Veg	r1i1p1f1	EC-Earth (2019d, 2019e, 2019f)
GFDL-ESM4	r1i1p1f1	Krasting et al. (2018a, 2018b), John et al. (2018)
IPSL-CM6A-LR	r1i1p1f1	Boucher et al. (2018a, 2018b, 2019a)
MCM-UA-1-0	r1i1p1f1	Stouffer (2019a, 2019b)
MPI-ESM1-2-LR	r1i1p1f1	Wieners et al. (2019a, 2019b, 2019c)
NorESM2-LM	r1i1p1f1	Seland et al. (2019a, 2019b)
NorESM2-MM	r1i1p1f1	Bentsen et al. (2019a, 2019b, 2019c)
SAM0-UNICON	r1i1p1f1	Park and Shin (2019a, 2019b)
UKESM1-0-LL	r1i1p1f2	Tang et al. (2019a, 2019b), Good et al. (2019)

Supplementary Table. 2. CMIP6 GCM ensemble model information. For a list of the model acronyms, see http://www.ametsoc.org/PubsAcronymList.

References

1. Bentsen, M., Oliviè, D. J. L., Seland, Ø., Toniazzo, T., Gjermundsen, A., Graff, L. S., … Schulz, M. (2019a). *NCC NorESM2-MM model output prepared for CMIP6 CMIP historical*. Version *20191108*. Earth System Grid Federation. https://doi.org/10.22033/ESGF/CMIP6.8040
2. Bentsen, M., Oliviè, D. J. L., Seland, Ø., Toniazzo, T., Gjermundsen, A., Graff, L. S., … Schulz, M. (2019b). *NCC NorESM2-MM model output prepared for CMIP6 CMIP piControl*. Version *20191108*. Earth System Grid Federation. https://doi.org/10.22033/ESGF/CMIP6.8221
3. Bentsen, M., Oliviè, D. J. L., Seland, Ø., Toniazzo, T., Gjermundsen, A., Graff, L. S., … Schulz, M. (2019c). *NCC NorESM2-MM model output prepared for CMIP6 ScenarioMIP*. Version *20191108.* https://doi.org/10.22033/ESGF/CMIP6.608
4. Boucher, O., Denvil, S., Levavasseur, G., Cozic, A., Caubel, A., Foujols, M.-A., … Cheruy, F. (2018a). *IPSL IPSL-CM6A-LR model output prepared for CMIP6 CMIP historical*. Version *20180803*. Earth System Grid Federation. https://doi.org/10.22033/ESGF/CMIP6.5195
5. Boucher, O., Denvil, S., Levavasseur, G., Cozic, A., Caubel, A., Foujols, M.-A., … Cheruy, F. (2018b). *IPSL IPSL-CM6A-LR model output prepared for CMIP6 CMIP historical*. Version *20190522*. Earth System Grid Federation. https://doi.org/10.22033/ESGF/CMIP6.5195
6. Boucher, O., Denvil, S., Levavasseur, G., Cozic, A., Caubel, A., Foujols, M.-A., … Lurton, T. (2019a). *IPSL IPSL-CM6A-LR model output prepared for CMIP6 ScenarioMIP ssp585*. Version *20190522*. https://doi.org/10.22033/ESGF/CMIP6.5271
7. Danabasoglu, G., Lawrence, D., Lindsay, K., Lipscomb, W., & Strand, G. (2019). *NCAR CESM2 model output prepared for CMIP6 CMIP piControl*. Version *20190320*. https://doi.org/10.22033/ESGF/CMIP6.7733
8. Danabasoglu, G. (2019a). *NCAR CESM2 model output prepared for CMIP6 CMIP historical*. Version *20190308*. https://doi.org/10.22033/ESGF/CMIP6.7627
9. Danabasoglu, G. (2019b). *NCAR CESM2 model output prepared for CMIP6 ScenarioMIP ssp585*. Version *20190308*. https://doi.org/10.22033/ESGF/CMIP6.7768
10. Danabasoglu, G. (2019c). *NCAR CESM2-WACCM-FV2 model output prepared for CMIP6 CMIP historical*. Version *20191120*. https://doi.org/10.22033/ESGF/CMIP6.11298
11. Danabasoglu, G. (2019d). *NCAR CESM2-WACCM-FV2 model output prepared for CMIP6 CMIP piControl*. Version *20191120*. https://doi.org/10.22033/ESGF/CMIP6.11302
12. Danabasoglu, G. (2019e). *NCAR CESM2-WACCM model output prepared for CMIP6 ScenarioMIP ssp585*.

Version *20191120*. https://doi.org/10.22033/ESGF/CMIP6.10115

13. Danabasoglu, G. (2019f). *NCAR CESM2-WACCM model output prepared for CMIP6 CMIP historical*. Version *20190808*. https://doi.org/10.22033/ESGF/CMIP6.10071
14. Danabasoglu, G. (2019g). *NCAR CESM2-WACCM model output prepared for CMIP6 CMIP piControl*. Version *20190320*. https://doi.org/10.22033/ESGF/CMIP6.10094
15. Danabasoglu, G. (2019h). *NCAR CESM2-WACCM model output prepared for CMIP6 ScenarioMIP ssp585*. Version *20190320*. https://doi.org/10.22033/ESGF/CMIP6.10115
16. Dix, M., Bi, D., Dobrohotoff, P., Fiedler, R., Harman, I., Law, R., … Yang, R. (2019a). *CSIRO-ARCCSS ACCESS-CM2 model output prepared for CMIP6 CMIP historical*. Version *20191108*. Earth System Grid Federation. https://doi.org/10.22033/ESGF/CMIP6.4271
17. Dix, M., Bi, D., Dobrohotoff, P., Fiedler, R., Harman, I., Law, R., … Yang, R. (2019b). *CSIRO-ARCCSS ACCESS-CM2 model output prepared for CMIP6 CMIP piControl*. Version *20191112*. Earth System Grid Federation. https://doi.org/10.22033/ESGF/CMIP6.4311
18. Dix, M., Bi, D., Dobrohotoff, P., Fiedler, R., Harman, I., Law, R., … Yang, R. (2019c). *CSIRO-ARCCSS ACCESS-CM2 model output prepared for CMIP6 ScenarioMIP ssp126*. Version *20191112*. https://doi.org/10.22033/ESGF/CMIP6.4319
19. EC-Earth. (2019a). *EC-Earth-Consortium EC-Earth3 model output prepared for CMIP6 CMIP historical*. Version *20200310*. Earth System Grid Federation. https://doi.org/10.22033/ESGF/CMIP6.4700
20. EC-Earth. (2019b). *EC-Earth-Consortium EC-Earth3 model output prepared for CMIP6 CMIP piControl*. Version *20200312*. Earth System Grid Federation. https://doi.org/10.22033/ESGF/CMIP6.4842
21. EC-Earth. (2019c). *EC-Earth-Consortium EC-Earth3 model output prepared for CMIP6 ScenarioMIP ssp585*. Version *20200312*. https://doi.org/10.22033/ESGF/CMIP6.4912
22. EC-Earth. (2019d). *EC-Earth-Consortium EC-Earth3-Veg model output prepared for CMIP6 CMIP historical*. Version *20200225*. Earth System Grid Federation. https://doi.org/10.22033/ESGF/CMIP6.4706
23. EC-Earth. (2019e). *EC-Earth-Consortium EC-Earth3-Veg model output prepared for CMIP6 CMIP piControl*. Version *20200226*. Earth System Grid Federation. https://doi.org/10.22033/ESGF/CMIP6.4848
24. EC-Earth. (2019f). *EC-Earth-Consortium EC-Earth3-Veg model output prepared for CMIP6 ScenarioMIP ssp585*. Version *20200226*. Earth System Grid Federation. https://doi.org/10.22033/ESGF/CMIP6.4914
25. Eyring, V., Bony, S., Meehl, G. A., Senior, C. A., Stevens, B., Stouffer, R. J., and Taylor, K. E. (2016). *Overview of the Coupled Model Intercomparison Project Phase 6 (CMIP6) experimental design and organization*. Geoscientific Model Development., 9, 1937–1958, https://doi.org/10.5194/gmd-9-1937-2016

26. Good, P., Sellar, A., Tang, Y., Rumbold, S., Ellis, R., Kelley, D., & Kuhlbrodt, T. (2019). *MOHC UKESM1.0-LL model output prepared for CMIP6 ScenarioMIP ssp585*. Version *20190627*. https://doi.org/10.22033/ESGF/CMIP6.6405
27. John, J. G., Blanton, C., McHugh, C., Radhakrishnan, A., Rand, K., Vahlenkamp, H., … Zeng, Y. (2018). *NOAA-GFDL GFDL-ESM4 model output prepared for CMIP6 ScenarioMIP ssp585*. Version *20190726*. https://doi.org/10.22033/ESGF/CMIP6.8706
28. Kiss, A. E., Hogg, A. M., Hannah, N., Boeira Dias, F., Brassington, G. B., Chamberlain, M. A.,. . . Zhang, X. (2020). *ACCESS-OM2: A Global Ocean-Sea Ice Model at Three Resolutions.* Geoscientific Model Development Discussions, 1–58. doi: https://doi.org/10.5194/gmd-13-401-2020
29. Krasting, J. P., John, J. G., Blanton, C., McHugh, C., Nikonov, S., Radhakrishnan, A., … Zhao, M. (2018a). *NOAA-GFDL GFDL-ESM4 model output prepared for CMIP6 CMIP historical*. Version *20190726*. https://doi.org/10.22033/ESGF/CMIP6.8597
30. Krasting, J. P., John, J. G., Blanton, C., McHugh, C., Nikonov, S., Radhakrishnan, A., … Zhao, M. (2018b). *NOAA-GFDL GFDL-ESM4 model output prepared for CMIP6 CMIP piControl*. Version *20180701*. https://doi.org/10.22033/ESGF/CMIP6.8669
31. Levitus, S.et al. (2012). *World ocean heat content and thermosteric sea level change (0–2000 m)*. 1955–2010.301 Geophysical Research Letters 39. doi: https://doi.org/10.1029/2012GL051106
32. Morice, C. P., Kennedy, J. J., Rayner, N. A., Winn, J. P., Hogan, E., Killick, R. E., . . . Simpson,I. R. (2021). *An updated assessment of near-surface temperature change from 1850: The HadCRUT5 data set.* Journal of Geophysical Research: Atmospheres,126(3), e2019JD032361.doi: https://doi.org/10.1029/2019JD032361
33. Park, S., & Shin, J. (2019a). *SNU SAM0-UNICON model output prepared for CMIP6 CMIP historical*. Version *20190323*. https://doi.org/10.22033/ESGF/CMIP6.7789
34. Park, S., & Shin, J. (2019b). *SNU SAM0-UNICON model output prepared for CMIP6 CMIP piControl*. Version *20190910*. https://doi.org/10.22033/ESGF/CMIP6.7791
35. Rong, X. (2019a). *CAMS CAMS_CSM1.0 model output prepared for CMIP6 CMIP historical*. Version *20190708*. https://doi.org/10.22033/ESGF/CMIP6.9754
36. Rong, X. (2019b). *CAMS CAMS_CSM1.0 model output prepared for CMIP6 CMIP piControl*. Version *20190729*. https://doi.org/10.22033/ESGF/CMIP6.9797
37. Rong, X. (2019c). *CAMS CAMS-CSM1.0 model output prepared for CMIP6 ScenarioMIP*. Version *20190729*. https://doi.org/10.22033/ESGF/CMIP6.11004
38. Seferian, R. (2018a). *CNRM-CERFACS CNRM-ESM2-1 model output prepared for CMIP6 CMIP*

historical. Version *20181206*. https://doi.org/10.22033/ESGF/CMIP6.7127

39. Seferian, R. (2018b). *CNRM-CERFACS CNRM-ESM2-1 model output prepared for CMIP6 CMIP piControl*. Version *20181115*. https://doi.org/10.22033/ESGF/CMIP6.4165
40. Seland, Ø. (2019a). *NCC NorESM2-LM model output prepared for CMIP6 CMIP historical*. Version *20190815*. https://doi.org/10.22033/ESGF/CMIP6.8036
41. Seland, Ø. (2019b). *NCC NorESM2-LM model output prepared for CMIP6 CMIP piControl*. Version *20190920*. https://doi.org/10.22033/ESGF/CMIP6.8217
42. Stouffer, R. (2019a). *UA MCM-UA-1-0 model output prepared for CMIP6 CMIP historical*. Version *20190731*. https://doi.org/10.22033/ESGF/CMIP6.8888
43. Stouffer, R. (2019b). *UA MCM-UA-1-0 model output prepared for CMIP6 CMIP piControl*. Version *20190731*. https://doi.org/10.22033/ESGF/CMIP6.8890
44. Swart, N. C., Cole, J. N. S., Kharin, V. V, Lazare, M., Scinocca, J. F., Gillett, N. P., … Sigmond, M. (2019a). *CCCma CanESM5-CanOE model output prepared for CMIP6 CMIP historical*. Version *20190429*. https://doi.org/10.22033/ESGF/CMIP6.10260
45. Swart, N. C., Cole, J. N. S., Kharin, V. V, Lazare, M., Scinocca, J. F., Gillett, N. P., … Sigmond, M. (2019b). *CCCma CanESM5-CanOE model output prepared for CMIP6 CMIP piControl*. Version *20190429*. https://doi.org/10.22033/ESGF/CMIP6.10266
46. Swart, N. C., Cole, J. N. S., Kharin, V. V, Lazare, M., Scinocca, J. F., Gillett, N. P., … Sigmond, M. (2019c). *CCCma CanESM5-CanOE model output prepared for CMIP6 ScenarioMIP ssp585*. Version *20190429*. https://doi.org/10.22033/ESGF/CMIP6.10276
47. Tang, Y., Rumbold, S., Ellis, R., Kelley, D., Mulcahy, J., Sellar, A., … Jones, C. (2019a). *MOHC UKESM1.0-LL model output prepared for CMIP6 CMIP historical*. Version *20190627*. https://doi.org/10.22033/ESGF/CMIP6.6113
48. Tang, Y., Rumbold, S., Ellis, R., Kelley, D., Mulcahy, J., Sellar, A., … Jones, C. (2019b). *MOHC UKESM1.0-LL model output prepared for CMIP6 CMIP piControl*. Version *20190827*. https://doi.org/10.22033/ESGF/CMIP6.6298
49. Tsujino, H. et al. (2018) *JRA-55 based surface dataset for driving ocean–sea-ice models (JRA55-do).* Ocean Modelling 130, 79–139. doi: https://doi.org/10.1016/j.ocemod.2018.07.002
50. Voldoire, A. (2019). *CNRM-CERFACS CNRM-ESM2-1 model output prepared for CMIP6 ScenarioMIP ssp585*. Version *20181115*. https://doi.org/10.22033/ESGF/CMIP6.4226
51. Wieners, K.-H., Giorgetta, M., Jungclaus, J., Reick, C., Esch, M., Bittner, M., … Roeckner, E. (2019a).

MPI-M MPI-ESM1.2-LR model output prepared for CMIP6 CMIP historical. Version *20190710*. https://doi.org/10.22033/ESGF/CMIP6.6595

52. Wieners, K.-H., Giorgetta, M., Jungclaus, J., Reick, C., Esch, M., Bittner, M., … Roeckner, E. (2019b). *MPI-M MPI-ESM1.2-LR model output prepared for CMIP6 CMIP piControl*. Version *20190710*. https://doi.org/10.22033/ESGF/CMIP6.6675
53. Wieners, K.-H., Giorgetta, M., Jungclaus, J., Reick, C., Esch, M., Bittner, M., … Roeckner, E. (2019c). *MPI-M MPI-ESM1.2-LR model output prepared for CMIP6 ScenarioMIP ssp585*. Version *20190710*. https://doi.org/10.22033/ESGF/CMIP6.6705
54. Ziehn, T., Chamberlain, M., Lenton, A., Law, R., Bodman, R., Dix, M., … Druken, K. (2019a). *CSIRO ACCESS-ESM1.5 model output prepared for CMIP6 CMIP historical*. Version *20191115*. https://doi.org/10.22033/ESGF/CMIP6.4272
55. Ziehn, T., Chamberlain, M., Lenton, A., Law, R., Bodman, R., Dix, M., … Druken, K. (2019b). *CSIRO ACCESS-ESM1.5 model output prepared for CMIP6 CMIP piControl*. Version *20191115*. https://doi.org/10.22033/ESGF/CMIP6.4312
56. Ziehn, T., Chamberlain, M., Lenton, A., Law, R., Bodman, R., Dix, M., … Ridzwan, S. M. (2019c). *CSIRO ACCESS-ESM1.5 model output prepared for CMIP6 C4MIP esm-ssp585*. Version *20191115*. https://doi.org/10.22033/ESGF/CMIP6.4252
